# Disabilities research in India

**DOI:** 10.4103/0019-5545.69252

**Published:** 2010-01

**Authors:** H. Chandrashekar, C. Naveen Kumar, N. R. Prashanth, P. Kasthuri

**Affiliations:** Department of Psychiatry, BMC and RI, Bangalore, India

**Keywords:** Disability, IDEAS, mental illness

## Abstract

The objective of this paper is to review all articles related to psychiatric disability that have been published in the Indian Journal of Psychiatry since its inception till date. We have also added up some more relevant literature in the area of mental disability of Indian psychiatric patients. Finally the article ends up with discussion related to challenges associated with mental disability, persons with Disability Act and future directions in the area of psychiatric disability.

## INTRODUCTION

In the past three decades, the concept of disability has shifted from individual impairment to a more social phenomenon. Thus disability is a complex phenomenon, reflecting an interaction between features of a person’s body and features of the society in which he or she lives. In this view, persons with disabilities are seen as being restricted in performing daily activities because of a complex set of interrelating factors, some pertaining to the person and some pertaining to the person’s environment and social/political arrangements. The social concept of disability introduces the notion that society has erected barriers, physical or attitudinal, which affect a person with disabilities. Consequently, government programs and policies have evolved to include fixing the environment (e.g., making buildings barrier-free) and providing income assistance or work-related supports to help persons with disabilities participate more fully in the community and the workplace. Even the World Health Organization (WHO) goes beyond a medical approach to take a much broader view of disability. It also recognizes the role environment plays in either facilitating functioning or raising barriers.[1]

It is a well accepted fact that mental illnesses are also associated with a significant disability. Nearly 31% of the world’s disability is accounted by mental disorders. It was found that five of the ten leading causes of disability worldwide are in the category of mental illnesses: major depression, alcohol dependence, schizophrenia, bipolar affective disorder and obsessive-compulsive disorder.[2] World health report 2001 by the WHO assessed the leading causes of disability using disability adjusted life years (DALY). Mental illnesses accounted for 25% of total disability and 16 percent of total burden.[3]

Psychiatric disorders, by virtue of their very nature, display different pattern of disabilities when compared to that of physical ailments. It is important to note that social and work related functioning are more important in those with mental illnesses. We also need to remember that mental disability in the form of apathy, amotivation, poor self care communication difficulties and poor inter personal skills are not visible unlike other disabilities, viz. blindness or locomotor disability. There are instances when disability benefits like bus passes were denied because they look physically strong. It is compounded by stigma and discrimination. It is in this context, measures of psychiatric disability have been designed.

Research initiatives in the area of psychiatric disability in India have focused more on schizophrenia. Attention has been given to two important issues: Development or modification of scales for assessments and secondly disability evaluation in persons with chronic psychiatric illnesses. Disability has been assessed in psychiatric patients in different settings such as in hospitals, in community and also in follow-up studies.[2] As early as in 1979, Wig *et al*.[4] constructed a scale to measure disability of Indian psychiatric patients. They found that psychotics (ICD-8) obtained significantly higher scores than neurotics and persons with greater personal disability accepted treatment more often than those with less personal disability scores.

A decade later, Thara *et al*.[5] modified the Disability Assessment Schedule (WHO DAS-II) by deleting certain items and regrouping the rest into four main areas of personal, social, occupational and global disability because the DAS II was not entirely culture-free. This modified instrument was developed, validated and called the Schedule for Assessment of Psychiatric Disability (SAPD). They also administered this to 30 patients, each of the three groups of psychoses, neurotics and diabetics. It was found that the SAPD effectively discriminated the psychotic group from the other two groups. The authors recommended this instrument for measurement of disability in out-patient psychiatric population.

Further, Thara and Rajkumar[6] followed up 68 schizophrenia patients prospectively for a period of six years using standardized instruments. Disability was assessed using the SAPD at the end of four, five and six years of follow-up. They found that the three-year course of disability tended to be stable without any fluctuations and that the highest disability was in the area of occupational functioning. Moreover, the disability was not related to the number of relapses. The authors noted that this could be due to the following factors: The cohort was closely followed up and well treated; all patients were started treatment early in the course of their illness.

Shankar *et al*.[7] reported the gender differences in disability among married patients with schizophrenia. The study sample included 30 married patients of both sexes. Disability was evaluated using the modified version of the Disability assessment schedule. Results indicated that women were more disabled than men, in contrast to the findings from literature elsewhere. Negative symptoms predominated among the factors associated with global disability in both sexes.

Srinivasa Murthy *et al*.[8] assessed the costs and effects of a community outreach program for untreated schizophrenia patients in a rural community. Hundred cases were recruited and provided appropriate psychotropic medication and psychosocial support. They also assessed every three months over one and a half years on symptomatology, disability, family burden, resource use and costs. Results showed that summary scores of disability along with psychotic symptoms and family burden were all reduced over the follow-up period. These were also accompanied by reductions in the costs of informal-care sector visits and family care-giving time.

Mohan *et al*.[2] undertook a tertiary hospital-based study to assess and compare disability using the IDEAS in patients with schizophrenia and obsessive-compulsive disorder. They included patients with only mild severity illness. Majority of the schizophrenia patients were from rural areas whereas most of the OCD patients were from an urban background. Patients in both groups had considerable global disability. Understandably, schizophrenia patients had significantly greater disability across all domains of IDEAS. Duration of illness had no effect on disability scores in schizophrenia patients, but it had a negative impact with respect to OCD.

Choudhry *et al*.[9] assessed some aspects of disability associated with seven psychiatric disorders: Schizophrenia, bipolar affective disorder, anxiety disorder, depression, obsessive-complusive disorder, dementia and mental and behavioral disorders due to the use of alcohol. Their aim was to: Evaluate the nature and quantity disabilities in the study groups, compare the degree of disability with the severity of the disorder, compare disability among various disorders and study the longitudinal stability of disability in the disorder groups. They assessed a total of 228 patients attending the outpatient department of Assam Medical College, Dibrugarh, India, between July 2003 and June 2004. Patients were initially diagnosed using the ICD-10 criteria. Further, for those who consented to participate in the study, interviewers administered schedule for clinical assessment for neuropsychiatry (SCAN). Severity of the disorders was assessed by applying commonly used rating scales for each specific disorder. Disability was assessed using the Indian Disability Evaluation and Assessment Scale (IDEAS). Patients were followed up at six and 12 months. Results showed that all seven disorders studied were associated with significant disability; schizophrenia being maximally disabling. The domains of disability varied across the various disorders studied. The disability tended to correlate with the severity of the disorders. Disability associated with alcohol use disorder and anxiety was comparable to disability due to obsessive-compulsive disorder. Though the follow-up rates were low, analysis of the available data showed that the disability across most disorders reduced at the end of six month follow-up and then tended to even out after that period.

Tharoor *et al*.[10] cross-sectionally compared the inter-episode quality of life (QOL) and disability of patients with remitted bipolar affective disorder (BAD) or recurrent depressive disorder (RDD) with and without co morbid chronic medical illness. Assessments were carried out on the four subgroups (20 patients in each). QOL assessment was carried out using the World Health Organization (WHO)-QOL-Brief kannada version and disability was assessed using the schedule for assessment of psychiatric disability (SAPD), which is the Indian modification of the WHO disability assessment schedule-II. In patients who had medical comorbidity, BAD patients were significantly more disabled in ‘social role’ domain when compared with RDD patients (*P* = 0.04); while RDD patients were significantly more disabled in the ‘home atmosphere’ domain (*P* = 0.001). In patients who did not have medical co morbidity, BAD patients were significantly more disabled in the overall behavior domain when compared to RDD patients (*P* = 0.002); while RDD patients were significantly more disabled in ‘assets and/or liabilities’ (*P* = 0.004) and home atmosphere (*P* = 0.001) domains. The QOL measures did not differ significantly between the two disorders. The authors concluded that the medical illnesses may have a role in increasing disability but less likely to have a significant impact on QOL in mood disorders when patients are euthymic.

Kumar *et al*.[11] assessed the prevalence and pattern of mental disability in a rural taluk of Karnataka district. This was a community-based cross-sectional study. One thousand subjects were randomly selected from four villages and IDEAS was administered. Overall prevalence of mental disability was 2.3%. Among the disabled, majority had mild disability, followed by severe, moderate and profound severity. All disabled subjects were previously diagnosed with one or the other mental disorder such as: Affective disorders, mental retardation, epilepsy, neurosis, schizophrenia, alcohol addiction.

Krishnadas *et al*.[12] measured cognitive dysfunction in 25 remitted schizophrenia patients attending a psychiatry unit of a general hospital. Remission was confirmed using the brief psychiatric rating scale (BPRS) and the scale for the assessment of negative symptoms (SANS). The following neurocognitive measures were used: PGI memory scale, Trail making tests A and B, Rey-Osterrieth complex figure test and frontal assessment battery. Disability was assessed using the IDEAS. Results showed that patients had considerable cognitive dysfunction across all measures. Moreover, the authors did not find a statistically significant relationship between cognitive dysfunction and disability scores.

Gururaj *et al*.[13] assessed the disability along with family burden and quality of life of moderately ill obsessive compulsive disorder (OCD) and compared those with that of schizophrenia patients of comparable severity. Disability was assessed using the WHO-DAS. Results showed that both groups were similar across most domains of disability. The authors concluded that OCD is associated with significant disability often comparable to schizophrenia. Thirthalli *et al*.[14] assessed disability in 182 community dwelling schizophrenia patients in Thirthalli taluk of Shimoga district of Karnataka using the Indian Disability Evaluation and Assessment Scale (IDEAS). Their aim was to compare disability of schizophrenia patients receiving continuous antipsychotic treatment with that among those who were not taking antipsychotics or taking irregular treatment. Results showed that patients on antipsychotics had significantly less disability across all domains and in total IDEAS scores. Treatment status predicted disability scores even after controlling for the effects of controlling factors like age, sex, education, socio-economic status, duration of illness and alcohol dependence/ harmful use. Different levels of exposure to antipsychotics were associated with different levels of disability. Though there was no randomization, this study was conducted with a naturalistic design. The two groups did not differ in any of the clinical or socio-demographic variables. The authors concluded that treatment with antipsychotics is associated with significantly less disability.

Thirthalli *et al*.[15] compared the course of disability in schizophrenia patients receiving antipsychotics and those remaining untreated in a rural community. Of the 215 patients identified, 58% were not receiving antipsychotics. Trained raters assessed the disability (IDEAS) in 190 of these at baseline and after one year. The course of disability in those who remained untreated was compared with that in those who received antipsychotics. Results showed that in patients who remained untreated, the mean disability scores remained unchanged, but in those who continued receiving treatment and in those antipsychotics were initiated, the scores showed a significant decline (indicating decrement in disability). The proportion of patients classified as ‘disabled’, declined significantly in the treated group, but remained the same in the untreated group. The authors concluded that treatment with antipsychotics in the community results in a considerable reduction in disability.

### Legislation for benefit of disabled persons

India being a signatory to the proclamation adopted in the meeting to launch Asian and Pacific decade of disabled persons 1993-2002 at Beijing from 1st to 5th December, 1992 had to enact a law for the benefit of the disabled. Hence the persons with disabilities (Equal opportunities, Protection of rights and Full Participation) act 1995 was passed in the parliament. Mental illness was included as one of the disabilities. Two important gazette notifications in this regard are:

1. Ministry of Social Justice and Empowerment Notification [Gazette no 49 dated18th Feb 2002] which states that
  - Mental illness has been recognized as one of the disabilities
  - Defined as any mental disorder other than mental retardation
  - A committee was constituted to prescribe guidelines for evaluation and assessment of mental illness (6^th^ Aug 2001).
2. Ministry of social Justice and Empowerment Notification [Gazette No. 49 dated 27^th^ Feb 2002]
  - Authorities to give certificate will be the medical board constituted by Govt [section (1) and (2) of section 73 of Person with Disability act 1995]
  - Certificate valid for 5 years or permanent
  - Director General of Health Services (DGHS) will be the final authority.

Although PDA 1995, defines mental illness as any mental disorder other than mental retardation and includes only persons suffering from more than 40% disability, not all mentally ill are disabled and hence the definition has to be changed. One proposed definition in this regard (amendments proposed to the PDA, 1995)[16] is ‘a disorder of mind that results in partial or complete disturbance in the person’s thinking, feeling and behavior, which may also result in recurrent or persistent inability or reduced ability to carry out activities of daily living, self-care, education, employment and participation in social life’. It is noteworthy in this context that the notification does not require any psychiatric diagnoses for the purpose of disability. Although a number of tools that measure psychiatric disability existed, there was a need to develop a simple instrument that led to scores and then percentages. Consequently, the Rehabilitation Committee of the Indian Psychiatric Society (IPS) came up with the Indian disability evaluation and assessment scale (IDEAS) in 2002. IDEAS was field tested in nine centers all over India and has now been gazetted by the Ministry of Social Justice and Empowerment, Government of India, as the recommended instrument to measure psychiatric disability (Thara 2005). According to the IPS, only patients with the following diagnoses as per ICD-10 or DSM criteria are eligible for disability benefits: Schizophrenia, OCD, bipolar disorder and dementia.[17]

Government benefits for the disabled include

- Travel concession in Railways: 75% concession to the disabled and an accompanying person
- Annual passes at concessional rates by the State Road Transport Corporations
- Monthly maintenance allowance: Rs 400/- (for persons with disability between 40 and 70%) and Rs 1000/- for those whose disability exceeds 70%
- Benefits under various welfare programs like the Rojgar yojanas
- Income tax benefits
- Family pension: This will be given to the disabled after death of parents
- Employment reservation: Three to five per cent jobs in Government are reserved for disabled; Government has also identified jobs for mentally ill in this sector. In this context, it may by noted that the education department in a certain state government has reserved five per cent of its posts for the disabled; of this, one per cent is exclusively reserved for people with mental illness and 1% (only group-D posts) is exclusively for persons with mild levels of mental retardation
- Encouragement of students/self employment.

However, the number of patients getting benefits under the disability act is very low because of many barriers as listed below.

### Challenges and barriers of disability in mental illness

Attempts to improve the fate of the mentally disabled, especially in developing countries like India, face many obstacles. Stigmatization and discrimination are factors that come in the way of mentally ill receiving full disability benefits. People may have preconceived notions about the mentally ill - that these people are lazy or dangerous. These will have important consequences on how individuals come to see themselves and lowering their self esteem (Self-stigmatization) which may worsen their disability. The levels of knowledge of mental illness do not correlate with discriminatory attitudes. Even a proportion of medical personnel who are well informed are not tolerant towards the mentally ill. Consequences of discrimination include increasing vulnerability to disability, magnifying the impact of illness, depriving care and treatment. There also exist many barriers for the disabled to access the due benefits. These include: Stigma, poor knowledge about the IDEAS, fear of Misuse of Certificates, discomfort to approach government hospitals, time constraints, rigid negative thinking about legal issues, denial of disability, and ‘outside’ pressure to issue disability certificates.

## FUTURE DIRECTIONS AND CONCLUSIONS

The following are a few suggestions:

- The disabled should demand benefits; we should remember that family has the prime responsibility to look after disabled and get the benefits due to them
- The voice of the disabled needs to be recognized by the government
- Strong encouragement and assistance needs to be given to people with mental disability and their representatives to form organizations
- Information regarding disability needs to be disseminated far and wide across the country
- The attitude of a professional needs to change
- Organized monitoring of disability services and benefits disbursed is needed
- Lacunae in mental health laws include need to periodically review existing legislation and plan amendments or bring in new legislation from time to time
- There needs to be more research on factors associated with disability and psychiatric disorders
